# Lipoma at an unusual site - an intrinsic laryngeal lipoma involving the aryepiglottic fold

**DOI:** 10.1259/bjrcr.20220013

**Published:** 2022-04-12

**Authors:** Lavanya Yegnaraman, Vinita Rathi, Kavita Goyal

**Affiliations:** 1 University College of Medical Sciences, Delhi, India

## Abstract

Lipomas are common tumours. However they are a rare occurrence in the larynx. They are an unusual cause for hoarseness of voice and may at times present acutely with respiratory distress due to significant airway compromise. The characteristic imaging features help to differentiate it from laryngeal malignancy and other common pathologies. We present a case of a laryngeal lipoma arising from the aryepiglottic fold, which was diagnosed on imaging and confirmed on histopathological examination after surgical excision. We also briefly discuss the differential diagnoses of this entity.

## Introduction

Lipomas are benign mesenchymal tumours which commonly present in the fourth to sixth decade. However, laryngeal lipomas are rare entities and constitute only 1 % of all lipomas.^1^ The presentation at this unusual location can create diagnostic confusion and imaging is essential to differentiate it from a laryngeal malignancy as clinical presentation can be similar. Prompt diagnosis at imaging also helps to allay the fears in a patient.

## Case report

### Clinical presentation

A 45-year-old gentleman, a non-smoker, with no known co-morbidities, presented to the outpatient department of otorhinolaryngology, with complaints of change in voice and difficulty in swallowing for one month. There was no history of pain in the throat, fever, breathlessness or stridor. On indirect laryngoscopy, there was a large submucosal swelling in the supraglottic region involving the right pyriform sinus and false vocal cord with medialisation of the right false cord. The true vocal cords were not seen. The provisional clinical diagnosis was a supraglottic laryngeal malignancy.

### Imaging findings

**Figure 1.**
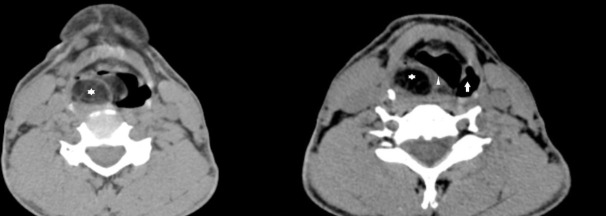
Axial unenhanced CT images reveal a well-defined, multilobulated lesion of fat attenuation (white star) seen involving the right aryepiglottic fold, which is thickened (white triangle) and obliterating the pyriform sinus. Thin septations are seen interspersed in the fat. The left pyriform sinus is normal (white block arrow).

**Figure 2.**
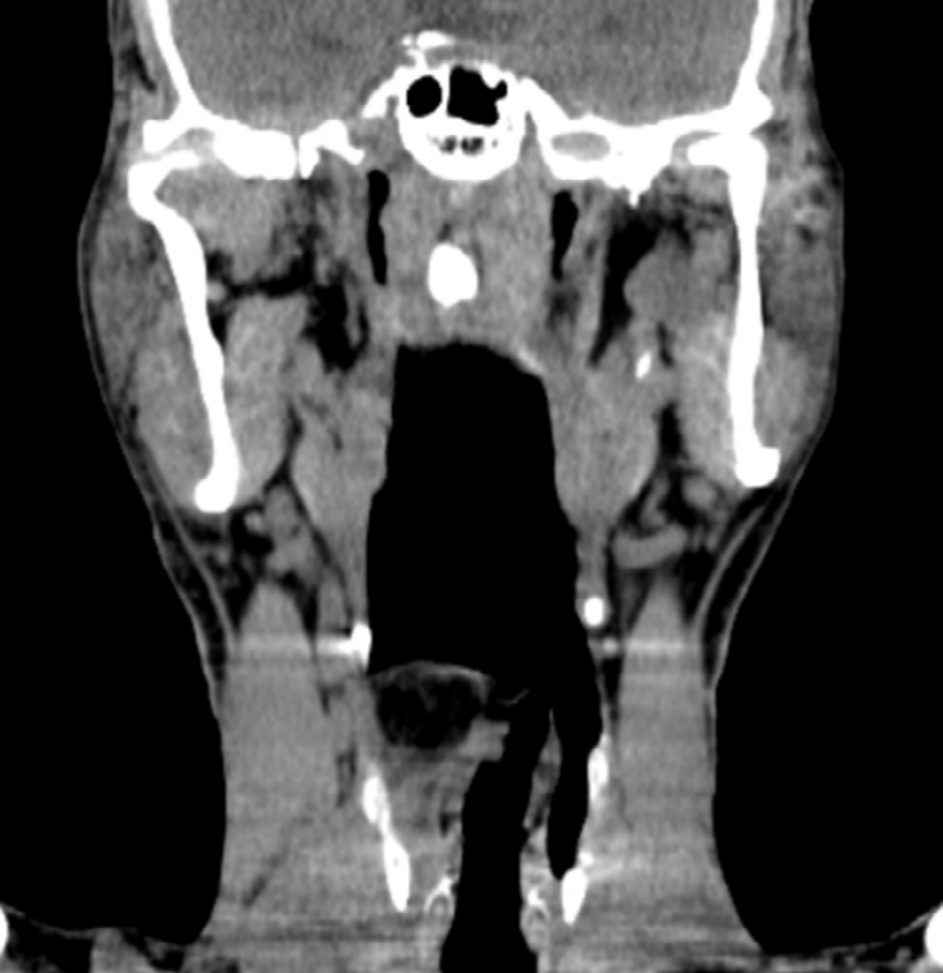
Coronal reformat image of unenhanced CT shows medialisation of the right false vocal cord (black arrow) and obliteration of the right pyriform sinus by the lipomatous mass.

**Figure 3.**
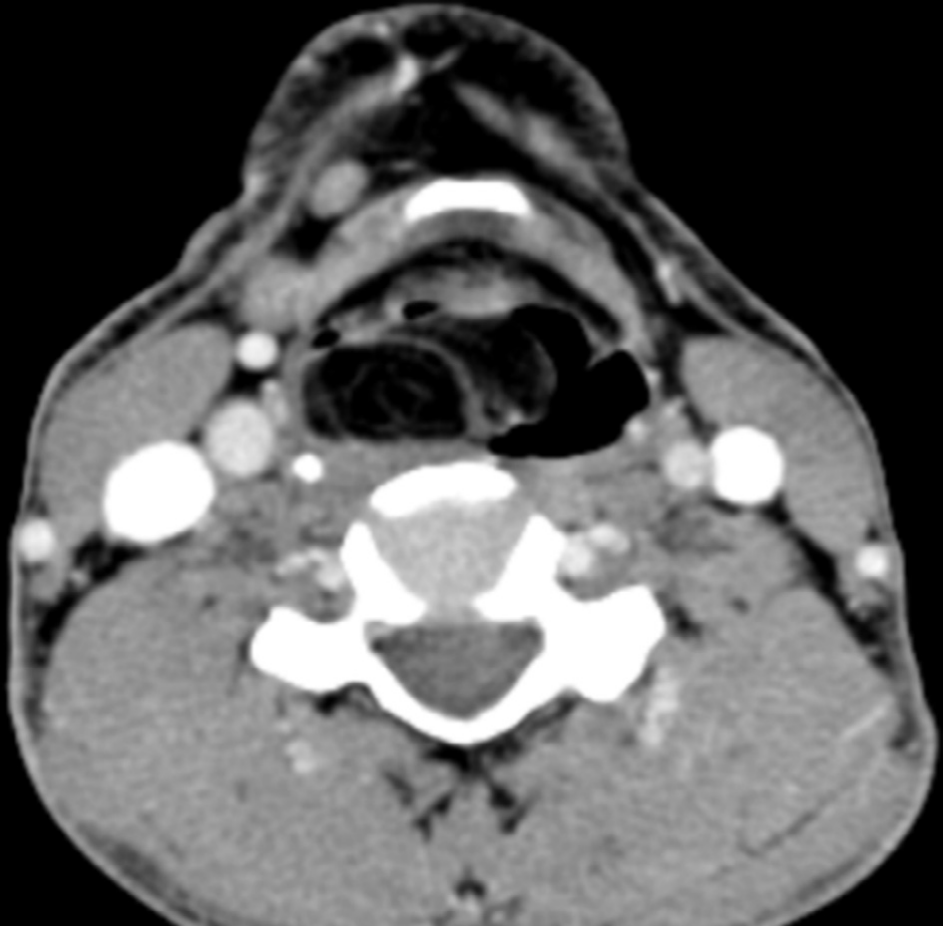
Contrast-enhanced CT image shows no enhancement in the mass or the septae

**Figure 4.**
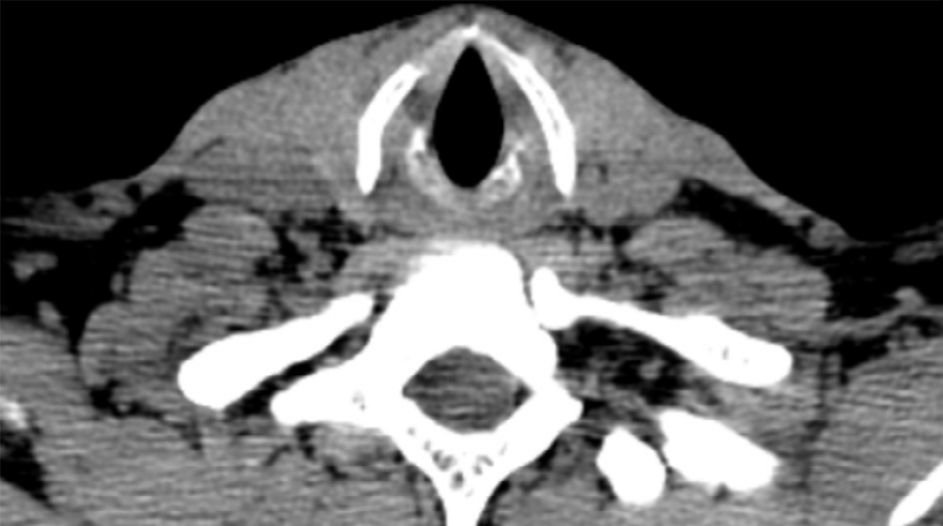
The true vocal cords and glottis are normal.

On CT scan, a multilobulated, well encapsulated, non-enhancing lesion of fat attenuation (−83HU) and size 1.7 × 3.1 x 1.9cm was seen involving the right aryepiglottic fold causing thickening at its base and obliteration of the right pyriform sinus. Multiple thin septations were seen within the lesion. Inferiorly, it extended up to the level of the right false vocal cord (Figures 1–3). The ventricles and the true vocal cords were normal (Figure 4).

**Figure 5.**
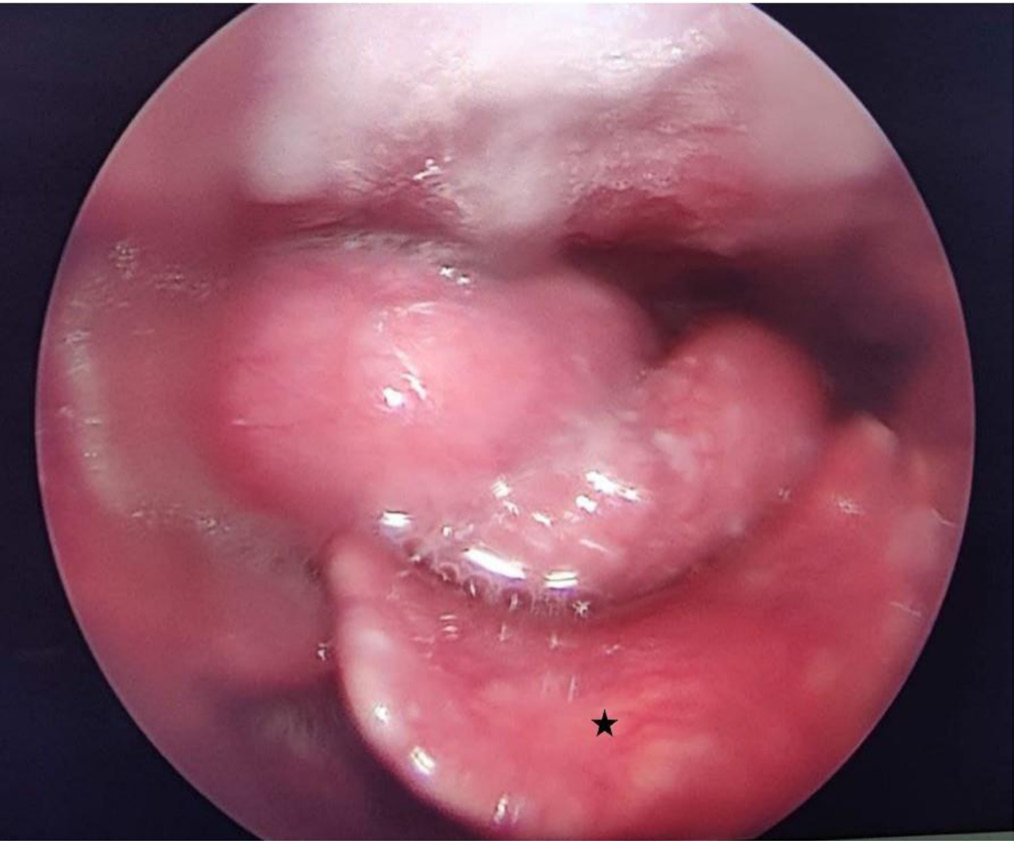
Image from video laryngoscopy showing a multinodular, smoothly marginated, soft tissue mass, posterior to the epiglottis (black star)

The patient underwent a video laryngoscopy which revealed a pedunculated fleshy mass originating from the right aryepiglottic fold, moving up and down with respiration; extending medially to obscure the view of endolarynx and laterally upto the medial wall of pyriform sinus (Figure 5).

### Management

The patient subsequently underwent direct laryngoscopy under general anaesthesia through a tracheostomy. A laryngoscopic excision of the mass via an intraoral approach was planned. However as the complete margins of the mass could not be visualised, the intraoral approach was abandoned and lateral pharyngotomy was undertaken. The mass, which was seen arising from right aryepiglottic fold, was excised along with the capsule. Histopathological examination of the specimen revealed mature adipose tissue and confirmed a simple lipoma.

### Follow up

Post-operative recovery of the patient was uneventful and he was asymptomatic at 3-month follow-up.

## Discussion

Although laryngeal lipomas are benign lesions, they can be dangerous if they cause airway obstruction. A few pedunculated lipomas arising from the aryepiglottic fold and epiglottis have been reported to cause death.^2^ They can also complicate intubation.^3^

Laryngeal lipomas are classified into intrinsic and extrinsic lipomas. The intrinsic forms are rarer and involve the aryepiglottic fold, false vocal cord and the epiglottis. The extrinsic forms involve the pyriform sinus and lingual surface of epiglottis.^1,4^

The differential diagnoses for a fatty lesion of the larynx include a well-differentiated liposarcoma, lipoblastoma and a hibernoma.^5^ However, imaging cannot reliably differentiate lipoma from a well-differentiated liposarcoma, lipoblastoma and hibernoma.^1^

Both lipoma and well-differentiated liposarcoma can present as smooth, well defined submucosal masses. Further imaging evaluation with MRI might help to differentiate between them. Complete suppression of signal on fat suppressed MR sequences and lack of enhancement of the septations favour the diagnosis of a benign lipoma. Sagittal reformations are useful in identifying the origin of a pedicle in pedunculated lipomas. Presence of haemorrhage and necrosis, and recurrence after complete surgical excision point towards a liposarcoma.^5^

A lipoblastoma, which generally occurs in infants and childhood, cannot be reliably differentiated from liposarcoma on imaging.^5^

A hibernoma is seen in the middle-aged population and originates from the metabolically active brown fat, owing to which it is FDG-avid on PET imaging. On CT, they have an intermediate attenuation between skeletal muscle and fat. On MRI, they are less intense than the subcutaneous fat and fail to suppress on fat suppression sequences.^1^

Other differentials include benign lesions such as laryngeal papilloma, chondroma and neurofibroma. Lipomas are distinguished from these possibilities by their characteristic imaging appearance - low attenuation on CT and high signal intensity on *T_1_*-weighted MRI.^4^

Endoscopic removal is advocated for pedunculated lipomas, whereas large (>2 cm) and un-pedunculated/submucosal tumours require an external surgical approach. As some of them could potentially be well differentiated liposarcomas, it is recommended to remove the whole of the tumour along with the pseudocapsule.^4^

Microscopically, lipomas are composed of mature adipose tissue with no cellular atypia.^6^ Histologically, lipomas could be simple (80% of the cases) or variants (20%) such as spindle cell lipomas, fibrolipomas, angiolipomas, myxolipomas and atypical lipomas.^2^ Even on histopathological examination, a differentiation between a well differentiated liposarcoma and a benign lipoma can be challenging. Hence, a long-term follow up is required in these patients to detect any recurrence which is seen in liposarcomas.^2,4^

## Learning points

1. Although laryngeal lipomas are rare entities, radiologists - especially trainees, need to be aware of this condition, as clinically they can mimic laryngeal malignancy and can rarely present in emergency with acute onset respiratory distress.
2. Imaging is important to assess the anatomy of the hypopharynx, larynx and subglottic region before surgery. These regions might be obscured by the mass at laryngoscopy.
3. Follow up is essential after surgical removal to detect the recurrence of liposarcoma, as histological differentiation between laryngeal lipoma and well-differentiated liposarcoma is unreliable.
